# Effects of educational technologies on the prevention and treatment of diabetic ulcers: A systematic review and meta-analysis*

**DOI:** 10.1590/1518-8345.6628.3945

**Published:** 2023-06-19

**Authors:** Jefferson Abraão Caetano Lira, Álvaro Sepúlveda Carvalho Rocha, Sandra Marina Gonçalves Bezerra, Paula Cristina Nogueira, Ana Maria Ribeiro dos Santos, Lídya Tolstenko Nogueira

**Affiliations:** 1 Universidade Federal do Piauí, Departamento de Enfermagem, Teresina, PI, Brasil.; 2 Universidade Estadual do Piauí, Centro de Ciências da Saúde, Teresina, PI, Brasil.; 3 Universidade de São Paulo, Escola de Enfermagem, Departamento de Enfermagem Médico-Cirúrgica, São Paulo, SP, Brasil.

**Keywords:** Diabetes Mellitus, Diabetic Foot, Diabetes Complications, Educational Technology, Systematic Review, Meta-Analysis, Diabetes *Mellitus*, Pie Diabético, Complicaciones de la Diabetes, Tecnología Educacional, Revisión Sistemática, Metaanálisis, Diabetes Mellitus, Pé Diabético, Complicações do Diabetes, Tecnologia Educacional, Revisão Sistemática, Metanálise

## Abstract

**Objective:**

to analyze the effects of educational technologies in the prevention and treatment of diabetic ulcers.

**Method:**

a systematic review conducted in seven databases, a bibliographic index, an electronic library and the Gray Literature. The sample consisted of 11 randomized controlled clinical trials. The synthesis of the results was descriptive and through meta-analysis.

**Results:**

the predominant educational technologies were training sessions and verbal guidelines, with soft-hard technologies standing out. When compared to usual care, the educational technologies presented a protective factor to prevent the incidence of diabetic ulcers (RR=0.40; 95% CI=0.18-0.90; p=0.03) and the certainty of the evidence assessment was low. The educational technologies also had a protective factor to prevent the incidence of lower limb amputations (RR=0.53; 95% CI=0.31-0.90; p=0.02) and certainty of the evidence was very low.

**Conclusion:**

soft-hard educational technologies such as structured verbal guidelines, educational games, lectures, theoretical-practical training sessions, educational videos, folders, serial albums and playful drawings, and hard technologies such as therapeutic footwear, insoles, infrared digital thermometer, foot care kits, Telemedicine app and mobile phone use, were effective for the prevention and treatment of diabetic ulcers, although more robust studies are required.

## Abstract (es)

**Objetivo:**

analizar los efectos de las tecnologías educativas en la prevención y tratamiento de la úlcera diabética.

**Método:**

revisión sistemática realizada en siete bases de datos, un índice bibliográfico, una biblioteca electrónica y literatura gris. La muestra estuvo compuesta por 11 ensayos clínicos controlados aleatorizados. La síntesis de los resultados fue descriptiva y mediante metaanálisis.

**Resultados:**

las tecnologías educativas predominantes fueron la capacitación y la orientación verbal, se destacaron las tecnologías blandas-duras. En comparación con la atención habitual, las tecnologías educativas demostraron ser un factor protector para prevenir la incidencia de úlcera diabética (RR=0,40; IC 95%=0,18-0,90; p=0,03) y la evaluación de certeza de evidencia fue baja. Las tecnologías educativas también demostraron ser un factor protector para prevenir la incidencia de amputación en miembros inferiores (RR=0,53; IC 95%=0,31-0,90; p=0,02) y la certeza de evidencia fue muy baja.

**Conclusión:**

tecnologías educativas blandas-duras, como orientación verbal estructurada, juegos educativos, clase expositiva, capacitación teórico-práctica, video educativo, *folder*, rotafolio educativo y dibujos lúdicos, y tecnologías duras, como calzado terapéutico, plantillas, termómetro infrarrojo digital, *kits* para el cuidado de los pies, aplicación de telemedicina y teléfono móvil, resultaron efectivas para la prevención y el tratamiento de la úlcera diabética, sin embargo, es necesario que se realicen estudios más robustos.

## Abstract (pt)

**Objetivo:**

analisar os efeitos das tecnologias educativas na prevenção e tratamento da úlcera diabética.

**Método:**

revisão sistemática conduzida em sete bases de dados, um índice bibliográfico, uma biblioteca eletrônica e na literatura cinzenta. A amostra foi constituída de 11 ensaios clínicos controlados randomizados. A síntese dos resultados foi descritiva e por meio de metanálise.

**Resultados:**

as tecnologias educativas predominantes foram os treinamentos e as orientações verbais, destacando-se as tecnologias leve-duras. Na comparação com o cuidado usual, as tecnologias educativas apresentaram fator de proteção para prevenção da incidência de úlcera diabética (RR=0,40; IC 95%=0,18-0,90; p=0,03) e a avaliação de certeza da evidência foi baixa. As tecnologias educativas também tiveram fator de proteção para prevenção da incidência de amputação em membros inferiores (RR=0,53; IC 95%=0,31-0,90; p=0,02) e a certeza da evidência foi muito baixa.

**Conclusão:**

as tecnologias educativas leve-duras, como orientações verbais estruturadas, jogos educativos, aula expositiva, treinamentos teórico-práticos, vídeo educativo, *folder*, álbum seriado e desenhos lúdicos, e as tecnologias duras, a exemplo do calçado terapêutico, palmilhas, termômetro digital de infravermelho, *kits* de cuidados com os pés, aplicativo de telemedicina e telefone móvel, foram efetivas para prevenção e tratamento da úlcera diabética, porém, estudos mais robustos são necessários.

**Highlights:**

**(1)** Educational technologies improved foot self-care.

**(2)** Educational technologies contributed to diabetic ulcer healing.

**(3)** Educational technologies were effective in preventing diabetic ulcers.

**(4)** Educational technologies presented a protective factor for amputation.

**(5)** It is recommended to use educational technologies in the prevention and treatment of diabetic ulcers.

## Introduction

Diabetic ulcers are a health problem resulting from chronic complications of diabetes *mellitus*, such as peripheral neuropathy and peripheral arterial disease. Peripheral neuropathy causes protective sensitivity loss, foot deformity, joint mobility limitation and abnormal biomechanical load on the feet, leading to the formation of calluses, subcutaneous hemorrhage and ulceration. Usually caused by atherosclerosis, peripheral artery disease is a risk factor for poor healing of diabetic ulcers and for lower limb amputation. Thus, diabetic ulcers are classified as neuropathic, ischemic or neuroischemic^(1)^.

Diabetic ulcers generate significant suffering and financial costs for the patients, in addition to overloading family members and health professionals and services, emphasizing the need for strategies that include elements of prevention, patient and team education, multidisciplinary treatment and rigorous monitoring^(2)^. Diabetic ulcer treatment should include relief of plantar pressure, removal of calluses, protection and drainage of blisters, treatment of fungal infections, intervention to accelerate healing, foot self-care guidelines and management of peripheral artery disease, in order to reduce ulceration complications such as delays in the healing process, presence of infections and lower limb amputations^(1)^.

In Spain, 44.1% of the patients with diabetes *mellitus* had neuroischemic ulcers, of which 20.3% were neuropathic and 20.3% were ischemic, with presence of infection as an aggravating factor in 41.4% of the cases^(3)^. The cumulative incidence of diabetic ulcers in Japan was 0.2% at 12 months, 2.4% at 60 months and 5.8% at 120 months, and most of these patients did not return for reevaluations^(4)^, highlighting the importance of care continuity and of implementing health education strategies to improve adherence to the therapy and prevent foot complications. A Brazilian study found that 1.9% of the patients with diabetes *mellitus* had diabetic ulcers, 59% had diabetic neuropathy, 69.6% were at risk of developing diabetic foot, and 86.3% of the patients reported never having undergone any clinical foot examination^(5)^.

Diabetic ulcers can be caused by trauma, inappropriate shoes, mycotic infections, nail problems, calluses, dry skin and cracks^(2,6)^. In addition to that, a study verified that patients with moderate knowledge about self-care practices were more likely to perform foot self-care, dry the interdigital spaces, moisturize the feet with creams and observe the presence of mycosis and ingrown toenails, when compared to those with insufficient knowledge^(7)^.

Diabetic foot is a complication that requires thorough monitoring and behavioral changes. Thus, educational technologies can be effective in controlling diabetes *mellitus*, stimulating the promotion of foot care and, in the long term, they can enable a reduction in costs, foot complications and amputations^(8)^. In this perspective, an educational intervention, with a practical skills session and foot care kit, reduced the risk factors for ulceration and improved the preventive behavior of foot self-care^(6)^.

Training sessions, verbal guidelines, leaflets, apps, videos and didactic games are educational technologies that can be used for the health education of professionals and patients with diabetes *mellitus*. Thus, structured education, callus removal, use of therapeutic footwear and physical exercises related to the feet and mobility are beneficial to improve modifiable risk factors for foot ulcerations^(9)^. In addition, the digital educational technology developed for nurses allows greater reach due to ease of access and to time, spatial and schedule flexibility, in addition to offering reduced costs. This educational strategy enables professional updating, qualification and training, contributing to the implementation of preventive interventions to reduce foot complications in patients with diabetes *mellitus* ^(8)^.

Educational technologies ease care management and, according to Merhy, they can be classified into soft, soft-hard and hard. Soft technologies consist of relationships such as welcoming, bonding and patient autonomy, through open dialog, qualified listening and group dynamics. Soft-hard technologies correspond to structured knowledge, such as serial albums, educational videos, pamphlets and posters. Hard technologies comprise material resources such as technological devices and registration forms^(10)^.

The diverse evidence about the effects of educational technologies to prevent the incidence of diabetic ulcers and foot complications is indispensable to guide the clinical practice and incorporate these technologies in the care of patients with diabetes *mellitus*, in order to improve care quality, comprehensive assistance, foot self-care and the patients’ quality of life and satisfaction levels, in addition to reducing costs, hospital admissions and non-traumatic lower limb amputations.

In view of the above, this systematic review and meta-analysis aimed at analyzing the effects of educational technologies in the prevention and treatment of diabetic ulcers.

## Method

### Type of study

This is a systematic review and meta-analysis prepared according to the recommendations set forth in the Cochrane collaboration, based on the following stages: 1) Elaboration and registration of the systematic review protocol; 2) Delimitation of the guiding question; 3) Definition of the eligibility criteria; 4) Search and selection of studies; 5) Data collection; and 6) Synthesis and presentation of the systematic review results^(11)^. The Preferred Reporting Items for Systematic Review and Meta-Analysis Protocols (PRISMA) guidelines were adopted to draft the systematic review and meta-analysis report^(12)^.

The review protocol was registered in the International Prospective Register of Systematic Reviews (PROSPERO), under number CRD42021287241^(13)^.

### Locus

The systematic review and meta-analysis was conducted in Teresina, capital city of Piauí, Brazil.

### Period

The systematic review and meta-analysis took place from January to October 2022.

### Research question

Formulation of the research question was delimited based on the PICOS acronym (P: Population or Patients; I: Intervention; C: Comparison; O: Outcomes; S: Study design), where P=Population (patients with diabetes *mellitus*), I=Intervention (educational technologies); C=Comparison (Control Group without receiving the intervention through educational technologies or receiving usual care), O=Outcomes (reduction in the incidence of ulcerations and diabetic ulcer complications) and S=Study design (randomized controlled clinical trials)^(14)^. In the comparison group, usual care consisted of the routine assistance offered by the service, such as consultations, verbal guidelines, clinical foot examination and use of therapeutic shoes. Delays in the healing process, presence of infections and lower limb amputations were considered as diabetic ulcer complications. The expected outcomes were reduction of ulceration and amputation in the lower limbs, in addition to improvement in diabetic ulcer healing. Thus, the following guiding question was formulated: Which are the effects of educational technologies on the prevention and treatment of diabetic ulcers in patients with diabetes *mellitus*? In this perspective, the care measures in the healing of ulcerations and the assistance provided to complications related to diabetic ulcers were considered as treatment.

### Eligibility criteria

The materials included were randomized controlled clinical trials that evaluated the effects of using educational technologies in the prevention and treatment of diabetic ulcers in patients with diabetes *mellitus*, without any time or language restrictions. The exclusion criteria were as follows: course completion papers, monographs, book chapters and materials that did not answer the guiding question. It is emphasized that randomized controlled clinical trials do not usually include the Gray Literature, that is, the one consisting of course conclusion papers, monographs and book chapters, representing an exclusion criterion in this study.

### Bibliographic survey and search strategy

For the bibliographic survey, databases, a bibliographic index and an electronic library were consulted, namely*:* Medical Literature Analysis and Retrieval System on-line (MEDLINE via PubMed^®^); Cumulative Index to Nursing and Allied Health Literature (CINAHL-EBSCO); Web of Science^TM^; Scopus; Embase; Cochrane Central Register of Controlled Trials (CENTRAL Cochrane); *Base de Dados em Enfermagem* (BDENF); the *Literatura Latino-Americana e do Caribe em Ciências da Saúde* (LILACS) bibliographic index, via *Biblioteca Virtual em Saúde* (BVS); and the Scientific Electronic Library Online (SciELO) library. The searches were carried out on the Journals Portal of *Coordenação de Aperfeiçoamento de Pessoal de Nível Superior* (CAPES), through access to the *Comunidade Acadêmica Federada* (CAFe) of the Federal University of Piauí.

The search strategies were developed by combining controlled descriptors and keywords, using the “*OR*” and “*AND*” Boolean operators according to the particularities of each database, index or library. In this sense, the Medical Subject Headings (MeSH) controlled vocabulary was consulted to select the search terms in the MEDLINE via PubMed^®^, Web of Science^TM^, Scopus and CENTRAL Cochrane databases, based on the following search strategy: ((((“diabetes mellitus”[MeSH Terms]) OR (“diabetes”[All Fields])) AND ((((((((((“educational technology”[MeSH Terms]) OR (“instructional technology”[All Fields])) OR (“multimedia”[MeSH Terms])) OR (“health education”[MeSH Terms])) OR (“educational intervention”[All Fields])) OR (“education, distance”[MeSH Terms])) OR (“communications media”[MeSH Terms])) OR (“instructional film and video”[All Fields])) OR (“audiovisual aids”[MeSH Terms])) OR (“teaching materials”[MeSH Terms]))) AND (((((“foot ulcer”[MeSH Terms]) OR (“plantar ulcer”[All Fields])) OR (“diabetic foot”[MeSH Terms])) OR (“foot ulceration”[All Fields])) OR (“foot ulcer diabetic”[All Fields]))) AND ((((((“clinical trial”[Publication Type]) OR (“clinical trial”[All Fields])) OR (“controlled clinical trial”[Publication Type])) OR (“controlled clinical trial”[All Fields])) OR (“randomized controlled trial”[Publication Type])) OR (“randomized controlled trial”[All Fields])). In the other databases, bibliographic index and electronic library, the search strategies used were similar, and the CINAHL Headings controlled vocabulary was used in CINAHL-EBSCO, Emtree in Embase and the Descriptors in Health Sciences (*Descritores em Ciências da Saúde*, DeCS) in BDENF, LILACS and SciELO. The keywords were selected from the suggestions of the controlled vocabularies and thorough prior in-depth readings on the theme.

In order to contemplate the Gray Literature, secondary searches were carried out in the following sources: clinical trial registry websites, such as ClinicalTrials.gov (National Institutes of Health, NIH, USA) and The Brazilian Clinical Trials Registry (via the ReBEC Platform), the CAPES theses and dissertations catalog, the University of São Paulo (USP) digital theses and dissertations library portal and the DART-Europe E-Theses Portal. In addition to that, the lists of final references of the randomized controlled trials included were manually analyzed in order to find important studies to be added.

Selection of the studies was initially developed by two reviewers, independently and blindly, following the stages indicated in the Preferred Reporting Items for Systematic Reviews and Meta-Analyses (PRISMA) 2020 statement, namely: identification, screening and inclusion^(12)^. The first step was to read the titles and abstracts. After applying the inclusion and exclusion criteria, the studies were eligible for the next stage, which consisted in reading the full-texts. The inclusion and exclusion criteria were applied again to reach the review sample. It is noted that, in the selection stage, there was disagreement between both reviewers regarding the inclusion of 12 studies; therefore, a third reviewer was called upon.

Subsequently, a manual search was performed in the references of the studies included. The Rayyan app was used to store, organize and remove duplicates and to blindly select the studies^(15)^. It is noted that the Rayyan app version used was the free one. In addition to that, the team of reviewers underwent prior training to learn how to use this tool in selection of the studies. Search and selection of the studies were carried out from January to May 2022.

### Data collection

Data extraction was by means of a form prepared by the authors of this review, containing the following items: authors; title of the study; year of publication; study locus; population and sample; information about the method; randomization; blinding; statistical analysis; follow-up time; type and classification of the educational technology; intervention group; control group; main results; and conclusion. Data collection was carried out independently by two reviewers, from June to August 2022. In relation to the items and/or divergent information, meetings were scheduled between the reviewers to discuss and resolve the discordant aspects until reaching consensus.

### Data treatment and analysis

To assess the risk of bias in the randomized controlled clinical trials, we used the Revised Cochrane risk-of-bias tool for randomized trials (RoB 2), proposed by the Cochrane collaboration, which has five domains: bias arising from the randomization process; bias due to deviations from intended intervention; bias due to missing outcome data; bias in measurement of the outcome; and bias in selection of the reported result^(16)^. This evaluation was performed by two independent reviewers. The doubts were discussed at the meetings, seeking consensus.

The synthesis of the results was performed descriptively and through meta-analysis. Thus, when performing the meta-analyses, the randomized controlled clinical trials were grouped into incidence of diabetic ulcers and lower limb amputations. The meta-analysis analysis model used was the random effect, performed using the Review Manager (RevMan) software, version 5.3, from the Cochrane collaboration.

The quality of the evidence assessment was elaborated according to the Grading of Recommendations Assessment, Development and Evaluation Working Group (GRADE)^(17)^. The evaluation was performed for each outcome analyzed. In the meta-analysis, the outcomes evaluated were the incidence values of diabetic ulcers and lower limb amputations regarding use of educational technologies. Certainty of the evidence can be assessed as high (strong confidence that the true effect is close to the estimated one), moderate (moderate confidence in the estimated effect), low (limited confidence in the estimate of the effect) and very low (very limited confidence in the estimate of the effect). The certainty of the evidence assessment was performed using the GRADEpro software^(18)^.

## Results

**Figure 1 -:**
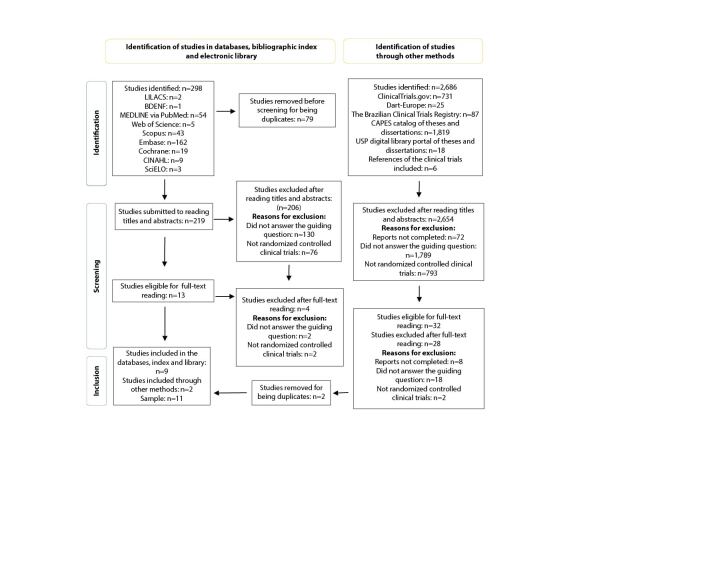
Flowchart corresponding to the selection process of the randomized controlled clinical trials included in the systematic review, adapted from the Preferred Reporting Items for Systematic Review and Meta-Analyses (PRISMA). Teresina, PI, Brazil, 2022

The bibliographic survey identified 2,984 studies: 298 in the databases, bibliographic index and electronic library and 2,686 in the Gray Literature. After removing the duplicates and applying the eligibility criteria, the sample resulted in 11 randomized controlled clinical trials^(19-20)^. Figure1 presents the detailed flowchart corresponding to the selection process of the studies included in the systematic literature review.

The randomized controlled clinical trials included a total of 3,115 participants^(19-20)^. In relation to the loci of the studies, there was prevalence of Brazil^(19,21)^, Norway^(22,23)^ and the United States^(24-25)^, with two studies each. The year of publication varied from 2000 to 2020 and the follow-up time, from one to 24 months. 23 soft-hard technologies, 16 hard technologies and one soft technology were identified in the intervention groups. The predominant educational technologies were training sessions in six studies^(22,25-26,27,23-20)^ and verbal guidelines, in five^(19-28,24,29-27)^. It was evidenced that, in five studies, the control groups did not receive any intervention through any educational technology^(19,22,21-20)^. The descriptive synthesis of the randomized controlled clinical trials included is presented in Figure2.

**Figure 2 -:** Synthesis of the randomized controlled clinical trials included (n=11). Teresina, PI, Brazil, 2022

Authors, year and locus	Sample/ Follow-up time	IG^*^/Type of educational technology (technology classification)	IG^†^/Type of technology (technology classification)	Main results
Cisneros (2010)^(19)^, Brazil	n=35/24 months	n=21/Verbal guidance through discussion of topics related to foot complications (soft-hard) and educational games (soft-hard).	n=14/Assistance routine offered by the service (did not use any educational technology).	Incidence of ulcerations: IG^*^: I^‡^=38.1% (8/21) CG^†^: I^‡^=57.1% (8/14) Recurrence of ulcerations: IG^*^: I^‡^=16.7% (1/8) CG^†^: I^‡^=83.3% (5/8)
Donohoe, et al. (2000)^(28)^, England	n=1,939/six months	n=981/Standardized leaflets (soft-hard) and structured verbal guidance (soft-hard).	n=958/Usual foot care, which included a practical visit (soft-hard) and an educational intervention on diabetic nephropathy (soft-hard).	Foot self-care: The attitudes toward foot care increased in both groups (IG^*^=3%; p<0.001 and CG^†^=1.8%; p<0.001) with no significant difference in the change between the groups (p=0.26).
Iversen, et al. (2020)^(22)^, Norway	n=182/12 months	n=94/Telemedicine app (hard) and mobile phone for guidance and communication between nurses from Primary Health Care and the specialized service (hard) and theoretical-practical training (soft-hard).	n=88/Standard care provided by the outpatient service, usually scheduled to occur every two weeks (did not use any educational technology).	Healing of diabetic ulcers: 82.1% of the patients had ulcer healing at 12 months in the IG^*^, and 76.9% in the CG^†^. There was no difference in the healing time between the groups. Incidence of amputations: IG^*^: I^‡^=5.1% (4/94) CG^†^: I^‡^=14.1% (11/88) Satisfaction: Satisfaction was similar for the IG^*^ and the CG^†^.
Lavery, et al. (2004)^(24)^, United States	N=85/six months	n=41/Diabetic foot education through verbal guidance (soft-hard), therapeutic shoes (hard), log book (hard) and portable infrared skin thermometer (hard).	n=44/Usual care, such as diabetic foot education (soft-hard) and therapeutic footwear (hard).	Incidence of diabetic ulcers: IG^*^: I^‡^=2.4% (1/41) CG^†^: I^‡^=15.9% (7/44) Incidence of amputations: IG^*^: I^‡^=0% (0/41) CG^†^: I^‡^=4.5% (2/44) Complications: There were 20% (n=9) of complications in the feet of the patients from the CG^†^ and 2% (n=1) of complications in those from the IG^*^ (p=0.01).
Lavery, et al. (2007)^(25)^, United States	n=173/15 months	n=59/Enhanced therapy: educational video (soft-hard), use of a digital infrared thermometer (hard), evaluation of the lower limbs (soft-hard), therapeutic insoles and shoes (hard) and logbook (hard). n=56/Structured foot examination: training for foot inspection (soft-hard), mirror (hard) and recording in a logbook (hard).	n=58/Standard therapy: evaluation of the lower limbs (soft-hard), educational video (soft-hard), therapeutic insoles and shoes (hard) and logbook (hard).	Incidence of diabetic ulcers: IG^*^ (enhanced therapy): I^‡^=8.5% (5/59) IG^*^ (structured foot exam): I^‡^=30.4% (17/56) CG^†^ (standard therapy): I^‡^=29.3% (17/58)
Liang, et al. (2012)^(26)^, China	n=62/24 months	n=31/Diabetes education lecture (soft-hard), training sessions through hands-on workshops (soft-hard), skills exercises (soft-hard) and foot care kit (hard).	n=31/Usual care, which consisted of two hours of diabetes education (soft-hard).	Incidence of ulcerations: IG^*^: I^‡^=0% (0/31) CG^†^: I^‡^=24.1% (7/31) Incidence of amputations: IG^*^: I^‡^=0% (0/31) CG^†^: I^‡^=6.9% (2/31) Foot self-care: There was a significant difference in knowledge and foot care in the IG^*^ participants (p<0.05).
Lincoln, et al. (2008)^(29)^, United Kingdom	n=172/12 months	n=87/Leaflets (soft-hard), handouts (soft-hard), illustrations (soft-hard), unstructured verbal guidelines in home visits (soft) and structured education, according to demand and by telephone (hard).	n=85/Leaflets (soft-hard) and unstructured and timely education (soft).	Incidence of ulcerations: IG^*^: I^‡^=41% (36/87) CG^†^: I^‡^=41% (35/85) Incidence of amputations: IG^*^: I^‡^=10% (9/87) CG^†^: I^‡^=11% (9/85) Foot self-care: The IG^*^ presented an apparent improvement in some foot care aspects.
Monami, et al. (2015)^(27)^, Italy	n=120/six months	n=60/Verbal guidelines on foot ulcer risk factors (soft-hard) and training through interactive practice with actions to reduce the foot ulcer risk factors (soft-hard).	n=60/Leaflet with some recommendations for the prevention of ulcers, according to local guidelines (soft-hard).	Incidence of ulcerations: IG^*^: I^‡^=0% (0/60) CG^†^: I^‡^=10% (6/60) Incidence of amputations: IG^*^: I^‡^=0% CG^†^: I^‡^=0% There was an improvement in the patients’ knowledge after the intervention (p<0.001).
Moreira, et al. (2020)^(21)^, Brazil.	n=109/one month	n=55/Illustrative and didactic folder (soft-hard), visual demonstrations (soft-hard), templates (hard), serial album (soft-hard), image projections (hard) and playful drawings (soft-hard).	n=54/Usual care, which consisted of routine care in the unit, with routine clinical follow-up (did not use any educational technology).	Reduced risk of foot complications: After 15 days of the intervention, there was statistical significance in relation to tissue injury, hairiness, hydration, perspiration, skin peeling, color after ten seconds of elevation, tissue perfusion, pedal and tibial pulses, edema, neuropathic symptoms and plantar pressure.
Smith-Strom, et al. (2018)^(23)^, Western Norway	n=182/12 months	n=94/Telemedicine app (hard), cell phone (hard) and theoretical-practical training (soft-hard).	n=88/Outpatient appointments every two weeks and, if necessary, additional monitoring (did not use any educational technology).	Healing of diabetic ulcers: 79.8% (n=75) had diabetic ulcer healing in the IG^*^ and 76.1% (n=67) in the CG^†^, with mean healing times of 3.4 and 3.8 months in the IG^*^ and CG^†^, respectively. Incidence of amputations: IG^*^: I^‡^=6.4% (6/94) CG^†^: I^‡^=14.8% (13/88) Patients’ satisfaction levels: Most of the patients in both groups reported high satisfaction with treatment and monitoring, with no differences between the groups.
Subrata, et al. (2020)^(20)^, Indonesia	n=56/three months	n=27/Skills training and motivational interview, which consisted of 50-minute sessions per week for three months and addressed the following topics: physical activity, medications, foot care, glycemic control, strengthening responsibilities, establishing roles and active involvement in care (soft-hard).	n=29/Usual care in diabetes (did not use any educational technology).	Healing of diabetic ulcers: The mean ulcer size in the IG^*^ decreased over time when compared to the CG^†^. Although not healing completely, the difference in ulcer size reduction was statistically significant between both groups (p<0.001).

*IG = Intervention Group; ^†^CG = Control Group; ^‡^I = Incidence

**Figure 3 -:**
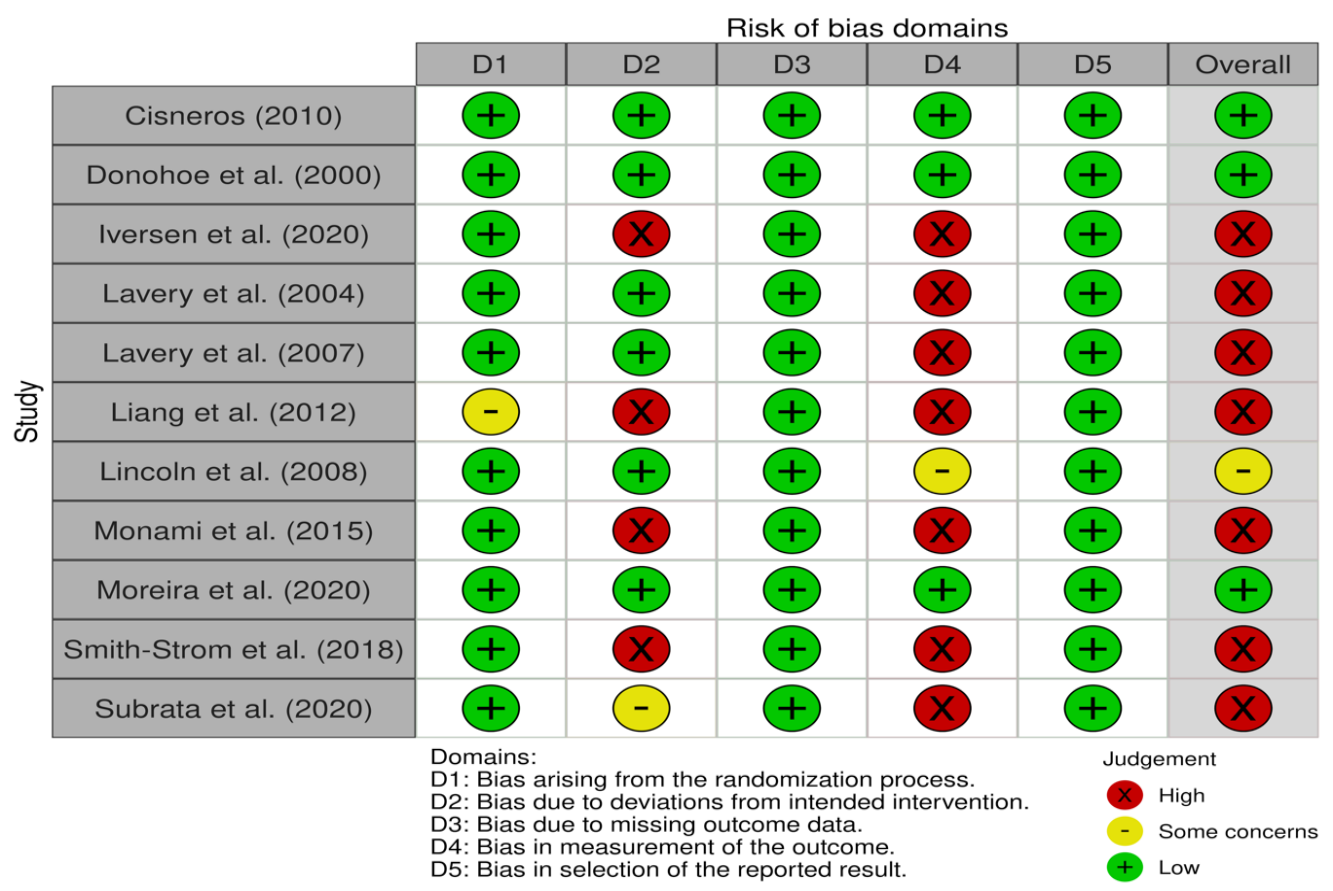
Risk of bias assessment of the randomized controlled clinical trials in each domain of the Revised Cochrane risk-of-bias tool for randomized trials (RoB 2). Teresina, PI, Brazil, 2022

Note: Figure generated in the Robvis tool app (2022)

Figure3 describes the risk of bias assessment using the RoB 2 tool, performed by domains for the 11 randomized controlled clinical trials included in the systematic review.

Of the 11 randomized controlled clinical trials, 27.3% (n=3) presented low risk of bias, 9.1% (n=1) had uncertain risk of bias, and 63.6% (n=7) were categorized as with high risk of bias. Seven studies^(22-26,27,23-20)^ were evaluated as with high risk in the bias domain in measurement of the results, as there was no blinding of the outcome evaluators. Four studies^(22,26,27,23)^ had a high risk in the bias domain due to deviations from the designated interventions, as a result of lack of blinding of the participants and the professionals who applied the intervention. One study^(29)^ was classified as having uncertain risk of bias, as it does not specify whether there was blinding of the evaluators. One study^(26)^ has some concern in the domain bias resulting from the randomization process, as randomization was performed but there are no details of the process in the method. One study^(20)^ presented some concern in the bias domain due to deviations from the designated interventions, as it did not clearly specify whether there was blinding of the professionals who applied the intervention.

In the meta-analysis, only randomized controlled clinical trials with similar characteristics were included, with regard to the interventions employed, in which the effects of the educational technologies were evaluated by the development of diabetic ulcers and lower limb amputations in the intervention and control groups. The Relative Risk (RR) was described in the last column of the forest plot, as shown in Figure4.

Figure 4 -Forest plots of the meta-analyses addressing the educational technologies *versus* usual care for the prevention of diabetic ulcers and lower limb amputations. Teresina, PI, Brazil, 2022.

**(A.1):**
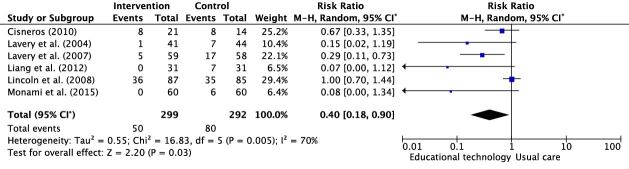
Educational technologies versus usual care for the prevention of diabetic ulcers

**(A.2):**
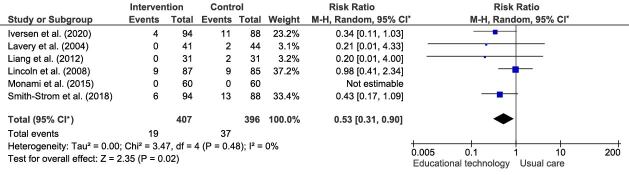
Educational technologies versus usual care for the prevention of lower limb amputations

Note: Graphs generated in the RevMan statistical program. ^*^CI = Confidence Interval


In Figure4 A.1, the meta-analysis, with six studies included^(19,24-27)^, which compared the educational technologies with usual care, evidenced that the use of educational technologies presented a protective factor for preventing the incidence of diabetic ulcers (RR=0.40; 95% CI=0.18-0.90; p=0.03). In Figure4 A.2, the meta-analysis, also with six studies^(22-24,26-27,23)^, indicates that the educational technologies have a protective factor to prevent the incidence of lower limb amputations, when compared to usual care (RR=0.53; 95% CI=0.31-0.90; p=0.02).

In Figure4 A.1, the Higgins inconsistency statistical test (I^2^) classified heterogeneity across the studies as substantial (I^2^=70%). In contrast, in Figure4 A.2, heterogeneity was indicated as not important (I^2^=0%).

Table1 presents the certainty assessment of the meta-analyses evidence according to the GRADE criteria. The estimated effect of the educational technologies for preventing the incidence of diabetic ulcers was RR=0.40, when compared to usual care, with low certainty of the evidence. The estimated effect of the educational technologies to prevent the incidence of lower limb amputations was RR=0.53, when compared to usual care, presenting very low certainty of the evidence.

**Table 1 -:** Synthesis of the certainty of the evidence assessment, according to the Grading of Recommendations Assessment, Development and Evaluation (GRADE). Teresina, PI, Brazil, 2022

Certainty of the evidence							Number of patients		Effect		
Number of studies	Type of study	Risk of bias	Inconsistency	Indirect evidence	Imprecision	Other considerations	Educational technology	Usual care	Relative (95% CI*)	Absolute (95% CI*)	Certainty
Incidence of diabetic ulcers/Educational technology versus usual care (follow-up: range from 6 months to 2 years)											
6	RCT^††^	Severe^‡^	Severe^§^	Not severe	Not severe	None	50/299 (16.7%)	80/292 (27.4%)	RR^ǁ^ = 0.40 (from 0.18 to 0.90)	164 less per 1,000 (from 225 less to 27 less)	⨁⨁◯◯ Low
Incidence of lower limb amputations/Educational technology versus usual care (follow-up: range from 6 months to 2 years)											
5	RCT^††^	Very severe^¶^	Not severe	Not severe	Severe**	None	19/347 (5.5%)	37/336 (11%)	RR^ǁ^ = 0.53 (from 0.31 to 0.90)	52 less per 1,000 (from 76 less to 11 less)	⨁◯◯◯ Very low

Note: Prepared in and extracted from the GRADEpro software

*CI = Confidence Interval; ^†^RCT = Randomized Clinical Trial; ^‡^The reason is that four studies present high risk of bias, with a weight of 45.4%; ^§^The reason for the assessment is that the Higgins inconsistency test (I²=70%) indicated substantial heterogeneity across the studies; ^ǁ^RR = Relative Risk; ^¶^The reason for the assessment is that four studies present high risk of bias, with a weight of 62.9%; **The reason for the assessment is that the effect estimate varies greatly

## Discussion

This study analyzed the effects of educational technologies on the prevention and treatment of diabetic ulcers, and the results evidenced that soft-hard educational technologies such as structured verbal guidelines, educational games, lectures, training sessions through workshops and interactive practice, educational videos, illustrative and didactic folders, serial albums and playful drawings, and hard technologies such as therapeutic footwear, insoles, digital infrared thermometer and foot care kits, contributed to reducing both the incidence of diabetic ulcers^(19,24-26,27)^ and the risk of foot complications^(21)^. In addition to that, the theoretical-practical training sessions, which are soft-hard technologies, and the Telemedicine apps and mobile phone use, which are classified as hard technologies, improved diabetic ulcer healing and reduced the incidence of lower limb amputations in the intervention groups^(22,23)^.

Educational technologies consist of knowledge enriched by human action, and are not merely about the construction and use of devices; they involve a systematic set of diverse scientific knowledge that enables planning, execution, control and monitoring of the educational process^(30)^. From this perspective, the particularities of the educational technologies explain the prevalence of soft-hard and hard technologies in the randomized controlled clinical trials included in this systematic review.

It was evidenced that eight randomized controlled clinical trials^(19-28,24-21)^ used soft-hard and/or hard educational technologies for the prevention of diabetic ulcers, which were effective in reducing the incidence of ulcerations in five studies^(19,24-26,27)^. On the other hand, three randomized controlled clinical trials^(22,23-20)^ used soft-hard and/or hard educational technologies in the treatment of diabetic ulcers, of which two^(22,23)^ found a considerable effect and recorded a higher percentage of total diabetic ulcer healing.

The soft-hard educational technologies were the most used in the prevention and treatment of diabetic ulcers in the intervention groups. Thus, a randomized controlled clinical trial carried out in Brazil, which used soft-hard technology, found that the implementation of educational technologies through a focus group and educational games addressing foot complications reduced the incidence of ulcerations and the recurrence of diabetic ulcers in the Intervention Group^(19)^. In Italy, a randomized controlled clinical trial, which in its Intervention Group used verbal guidelines on the risk factors for foot ulcerations and interactive practice, classified as soft-hard technologies, presented a significant effect in reducing the incidence of diabetic ulcers, as the Intervention Group had an incidence of 0% and the Control Group, 10%. In addition, they contributed to lowering the Body Mass Index and glycated hemoglobin, reinforcing that brief and low-cost educational technologies can reduce the incidence of foot ulcerations in patients with diabetes *mellitus*, in addition to being more likely to be applied in the routine clinical practice^(27)^.

Through a theoretical-practical approach, workshops and interactive practice, classified as soft-hard technologies, the training sessions proved to be effective in the prevention and treatment of diabetic ulcers and were the most prevalent educational technologies in six studies^(22,25-26,27,23-20)^. In Norway, through theoretical-practical training and Telemedicine, respectively classified as soft-hard and hard technologies, the educational technologies improved diabetic ulcer healing and reduced the number of amputations, as 82.1% of the patients in the Intervention Group presented ulcer healing in 12 months, with 5.1% incidence of amputations in the Intervention Group and 14.1% in the Control Group. In addition to that, this intervention increased the confidence of Primary Health Care nurses, who improved their skills in treating wounds, enabling a more comprehensive care for diabetic ulcers^(22)^.

The incidence of diabetic ulcers was estimated in six randomized controlled clinical trials^(19,24-27)^. Based on the meta-analysis, it was evidenced that the educational technologies presented a protective factor for preventing the incidence of diabetic ulcers, emphasizing the importance of using these resources in the assistance provided to patients with diabetes *mellitus*. In a prospective cohort study, the cumulative incidence of diabetic ulcers was 5.6% in two years, with the following risk factors for ulcerations: previous history of ulcerations or amputations, insulin consumption, distal neuropathy and foot deformity^(31)^. This emphasizes the need for care continuity to control the risk factors and for educational technologies aimed at preventing complications in patients with diabetes *mellitus*.

In this meta-analysis, the home-based educational session conducted in the United Kingdom with illustrations of injuries on the feet and a handout, classified as soft-hard technologies, did not present any statistically significant difference between the intervention and control groups regarding the prevention of diabetic ulcer incidence; however, there was an improvement in foot care behaviors in the Intervention Group in relation to checking the shoes before wearing them, daily foot washing and use of moisturizing creams^(29)^. On the other hand, a randomized controlled clinical trial carried out in the United States, which used enhanced therapy through educational video (soft-hard technology) associated with the use of therapeutic insoles and shoes (hard technologies), foot reevaluation (soft-hard technology) and use of a portable infrared thermometer to measure foot temperature (hard technology), identified a protective effect of this intervention for the prevention of diabetic ulcers, as there was a four-fold decrease in the risk of developing foot ulcers, with 29.3% incidence of ulcerations in the usual care group and 8.5% in the enhanced therapy group^(25)^.

In China, through lectures, practical workshops and skills exercises, which are soft-hard technologies, and the distribution of foot care kits, which included nail clippers, foot cream, mono-filament 10 g, thermometer to measure temperature of the water to wash the feet, pieces of cotton with alcohol and a mirror, which correspond to the hard technologies, the educational technologies had a significant effect in preventing the incidence of diabetic ulcers and amputations, in addition to the participants increasing their knowledge and foot care. Furthermore, the patients in the Control Group were nearly 24 times more likely to develop foot ulcers. This educational program asked the patients to perform daily foot care with the help of a mirror for foot inspection and invited at least one family member to participate in the classes and help the patients, which ensured more effective home-based foot care^(26)^.

Foot complications increase the likelihood of ulcerations, infections and amputations in people with diabetes *mellitus*. In this sense, a randomized controlled clinical trial, whose intervention consisted of verbal guidelines, which are soft-hard technologies, use of therapeutic shoes and an infrared thermometer, which are hard technologies, found that the patients in the Control Group had 10.3% more risks of developing some foot complication, with no statistical difference in terms of quality of life between the groups. In addition to that, the Control Group had seven ulcers and two Charcot fractures, with two patients developing infection and requiring amputation, whereas the Intervention Group had one ulcer and no amputations, highlighting that home-based self-monitoring of daily foot temperature, associated with health education and use of appropriate footwear, is an adjuvant tool for the prevention of diabetic ulcers and foot amputations^(24)^.

In a systematic review with meta-analysis, thermometry had a protective effect when compared to standard toe care to prevent the incidence of diabetic ulcers (RR=0.53; 95% CI=0.29-0.96; p=0.03), and the authors encourage managers, public health services, professionals, patients, family members and caregivers to implement this preventive technique by monitoring plantar temperature using infrared thermometers, both in the clinical and home contexts^(32)^. In this systematic review and meta-analysis, two randomized controlled clinical trials used thermometry associated with educational interventions^(24-25)^, which may have enhanced the effect of the educational technologies for the prevention of diabetic ulcers.

The incidence of lower limb amputations was estimated in six randomized controlled clinical trials and, in the meta-analysis, the educational technologies presented a protection factor to prevent amputations^(22-24,26-27,23)^. In a randomized controlled clinical trial, which employed Telemedicine in the community, classified as a hard technology, the incidence of amputations was 6.4% in the Intervention Group and 14.8% in the Control Group^(23)^. However, a study that used soft-hard technologies through a 30-minute face-to-face class and a 90-minute interactive practice on risk behaviors did not record the incidence of amputations between the control and intervention groups, which can be justified due to the brief 6-month follow-up period^(27)^.

Non-traumatic lower limb amputations are recurrent complications in patients with diabetes *mellitus*, generate increased costs for health services, extend the hospitalization times, reduce quality of life, exert impacts on mental health and affect the patients’ productive lives. Thus, foot care management, which includes health education, should value holistic care, accessibility, loyalty and care longitudinality. In this assumption, an education and continuous foot care treatment program in Spain detected that, of the total of 33 diabetic ulcers, 17 evolved to amputation and 16 were in patients who did not adhere to the program^(33)^.

In this perspective, diabetic foot complications are a public health problem due to the increase in the number of patients with diabetes *mellitus*, the increased life expectancy of the population and the growth of associated comorbidities. However, the expansion of the assistance provided, which includes both early intervention in patients with diabetic ulcers to avoid gangrene and appropriate treatments such as performing the necessary vascular procedures and mandatory education on foot care, can lead to a reduction in the number of lower limb amputations^(34)^.

Three randomized controlled clinical trials addressed the effect of educational technologies on diabetic ulcer healing^(22,23-20)^. Although the educational technologies employed, which were soft-hard and hard, did not exert any statistically significant effect on reducing the ulcer healing times^(22,23)^, there was a reduction in the size of the ulcers^(20)^, with 82.1% of the patients presenting ulcer healing in the Intervention Group and 76.9% in the Control Group at 12 months. This reinforces that educational technologies should also be used in the diabetic ulcer treatment stage^(22)^.

The effect of educational technologies on foot self-care was verified in four randomized controlled clinical trials^(28,26-29,21)^. Even without significant differences in behavioral changes (p=0.26), the attitudes regarding foot self-care increased in both groups^(28)^. In addition to that, an educational intervention for foot self-care, through an operative group which used soft-hard and hard technologies, had a significant effect in the treatment group after seven days (p<0.001) and 15 days (p<0.001), when compared to the Control Group, in relation to the reduction of the risks for foot complications, such as an improvement in the preservation of the skin and annexes, tissue perfusion, pulses, edema and plantar pressure distribution. This evidences that systematized educational interventions with brief follow-up periods are also effective^(21)^.

Thus, to enhance the effect, health education should reduce language barriers and involve the patients in their own care plan to raise awareness about the disease and prevent complications, as most patients are unaware of the severity of these complications and follow negligent practices in the long term, due to low education and risky cultural practices. Despite the challenges, health education is a responsibility of professionals, who must use every opportunity to provide specific education, even combining the types of educational technologies available, with the objective of improving the skills of patients with diabetes *mellitus* in foot self-care^(35)^.

An integrated care project, which included timely referral, weekly virtual clinic, healthy lifestyle support, community nurse training, app delivery and personalized educational support, increased engagement in education from 5% to 71% of those newly diagnosed with diabetes *mellitus*, in addition to reducing the incidence of major amputations from 13 to three procedures *per* 10,000 patients a year and of minor amputations from 26 to 18 procedures *per* 10,000 patients a year. This care model also significantly reduced the daily occupation of beds by people with diabetes *mellitus* in a district general hospital^(36)^. In line with this systematic review, when associated with better structuring of the care network and professional training, educational technologies are more effective in reducing foot amputations and hospitalization due to complications arising from diabetes *mellitus*.

Regarding the satisfaction levels provided by the educational technologies, both randomized controlled clinical trials that evaluated this outcome concluded that there was no statistically significant difference between the intervention and control groups^(22,23)^. However, the concern with satisfaction in the development of educational technologies is essential, as it influences the participants’ adherence to the intervention proposed.

The randomized controlled clinical trials included did not measure the costs of the educational technologies for the prevention and treatment of diabetic ulcers. Thus, the studies pointed out the need to carry out surveys comparing the costs of the educational and monitoring programs implemented with usual care, as it is expected that, in the long term, these interventions will present better cost-effectiveness, cost-benefit and cost-efficacy ratios in preventing foot complications and, consequently, reduce expenditure in health services and improve the quality of life of patients with diabetes *mellitus* ^(24-25,27)^.

In relation to the limitations of this systematic review, the reduced number of randomized controlled clinical trials on the effects of educational technologies on the treatment and incidence of diabetic ulcers stands out, in addition to the number of studies with a high risk of bias, which contributed to the lower certainty of the evidence.

The results of this systematic review may contribute to expanding the use of educational technologies in the care of patients with diabetes *mellitus*. In addition to that, this scientific evidence will assist health professionals in choosing the most assertive type of educational technology for the prevention and treatment of diabetic ulcers in the clinical practice.

## Conclusion

Soft-hard educational technologies such as structured verbal guidelines, educational games, lectures, training through workshops and interactive practice, educational video, illustrative and didactic folders, serial albums and playful drawings, and hard technologies such as therapeutic footwear, insoles, digital infrared thermometer and foot care kits, exerted a positive effect on the prevention of diabetic ulcers and helped reduce the incidence of ulcerations and the risk of foot complications, in addition to enabling improvements in foot care. In relation to the treatment, both the soft-hard technologies through theoretical-practical training sessions, and the soft technologies such as Telemedicine apps and use of mobile phones, contributed to the evolution of diabetic ulcer healing, standing out as useful strategies in foot care management in patients with diabetes *mellitus*.

The meta-analysis results indicated that the educational technologies presented a protective factor for preventing the incidence of diabetic ulcers, with substantial heterogeneity across the studies and a low certainty of the evidence assessment, highlighting that, in further research studies, there may be a change in the estimate of the effect. In addition to that, the educational technologies had a protective factor to prevent the incidence of lower limb amputations, when compared to usual care. Heterogeneity was indicated as not important, and certainty of the evidence was assessed as very low.

In view of this, the use of educational technologies is recommended, especially soft-hard and hard, in the prevention and treatment of diabetic ulcers to reduce complications such as non-traumatic lower limb amputations, in addition to conducting more robust and well-designed randomized controlled clinical trials at different care levels for patients with diabetes *mellitus*, which would later allow developing systematic reviews in different care contexts, with a view to reducing the risk of bias and inconsistencies, as well as improving homogeneity of the studies and certainty of the evidence, in order to incorporate those educational technologies that proved to be effective in foot care.
